# Experimental investigation of radiobiology in head and neck cancer cell lines as a function of HPV status, by MTT assay

**DOI:** 10.1038/s41598-018-26134-9

**Published:** 2018-05-17

**Authors:** Paul Reid, Puthenparampil Wilson, Yanrui Li, Loredana G. Marcu, Alexander H. Staudacher, Michael P. Brown, Eva Bezak

**Affiliations:** 1 School of Health Sciences, University of South Australia, Adelaide, Australia; 2 School of Engineering, University of South Australia, Adelaide, Australia; 3 Department of Medical Physics, Royal Adelaide Hospital, Adelaide, Australia; 4 School of Physical Sciences, University of Adelaide, Adelaide, Australia; 5 Faculty of Science, University of Oradea, Oradea, 410087 Romania; 6 Translational Oncology Laboratory, Centre for Cancer Biology, SA Pathology, University of South Australia, Adelaide, Australia; 7 School of Medicine, University of Adelaide, Adelaide, Australia; 8 Cancer Research Institute, University of South Australia, Adelaide, Australia; 9 Cancer Clinical Trials Unit, Royal Adelaide Hospital, Adelaide, Australia

## Abstract

Head and neck cancers (HNCs) are aggressive epithelial tumours frequently treated using radiation. HNC biology shows distinctions dependent on the oncologic involvement of the human papilloma virus (HPV). Clinically, HPV positive HNCs respond better to radiotherapy but few *in vitro* data demonstrate radiobiological differences explaining differences in clinical outcomes. This pilot study examined radiobiological responses to irradiation and subsequent regeneration in two HNC cell lines (HPV positive and negative). A novel approach was taken to develop generational cultures of HNC cell lines, UM-SCC-1 (HPV negative) and UM-SCC-47 (HPV positive). MTT assays were used to determine surviving metabolic activity as a function of dose following 6 MV X-ray irradiation. Parallel cultures surviving 4 Gy irradiation (not analysed) were re-cultured and passaged to develop subsequent generations which were re-irradiated and analysed for generational change in radiation response. Second and 3rd generations of UM-SCC-1 showed decreasing metabolic activity with dose but little difference was evident in surviving fractions between these generations. Significantly lower metabolic activity in the 3rd generation at <6 Gy, compared to the 2nd generation, showed UM-SCC-47 becoming progressively more radiosensitive. HPV positive UM-SCC-47 showed generational progression in radiosensitisation not seen in the HPV negative UM-SCC-1.

## Introduction

Head and neck cancers are typically aggressive epithelial tumours arising in the mucosae lining the mouth, nose, pharynx and larynx. More than 90% of these cancers are squamous cell carcinoma (HNSCC)^1,2^. Aetiological factors linked to the incidence of HNSCC are carcinogens such as tobacco, alcohol and betel quid. In developed countries, an increasing incidence of oropharyngeal HNSCC results from infection with the human papilloma virus (HPV). The prevalence of this form of HNSCC has resulted in the status classification of HNSCC as HPV positive or negative^3,4^. The global incidence for HNSCC is around 680,000 new cases annually and the survival rate of around 50% has changed little over the last few decades^5–7^. Although primary recurrence of HNSCC is the most frequent cause of mortality, distant metastasis is also an important cause of cancer specific death^6,8,9^. HNSCCs are often invasive cancers, involving surrounding vital tissue which may preclude surgical resection of the entire tumour. A multidisciplinary treatment approach is frequently required in which radiotherapy plays a prominent role^10^. Prescribed radiation doses, with intent to cure, are delivered in fractionated schedules, typically over 6–7 weeks. Complicating the prospect of tumour control are the responsive changes seen among cancer cells to radiation that can alter the parametrics by which the therapy has been prescribed^11,12^. Radiation induced changes in radiosensitivity can result quickly between the first and last treatment fractions^13^.

Clinical studies show HNSCC responds differently to radiotherapy, dependent on HPV status, such that radiotherapy exerts better tumour control in HPV positive patients than in the HPV negative patients. Consequently, a HPV positive status is taken to be a favourable prognostic indicator^14^. Treatment recommendations for HNSCC however, generally do not distinguish between the HPV statuses and this may partially result from a current lack of explanation for the difference seen in clinical outcomes^15,16^. Understanding radiobiological responsiveness of cancer cells treated at therapeutic dose levels, and radiation induced changes with fractionation, is critical in the application of radiotherapy for tumour control and in the minimisation of patient morbidities. Investigating the observed differences in treatment outcomes, based on the HPV status of disease, is clinically relevant and may help to identify features of the HPV negative disease that make it more refractory.

This study investigated specific radiobiological responses, as a function of dose, in 2 HNSCC cell lines re-cultured following repeated X-ray irradiation. A novel approach was taken whereby cell line cultures were re-assayed for metabolic activity in surviving fractions following previous radiation exposures and passage^17^. This identified ‘generational’ change in the metabolic survival of each cell line after exposure to a range of X-ray doses using standard MTT assay^18^. Generational changes in metabolic activity were also compared between cell lines of different HPV status. This approach results in the development of multiple concurrent cultures of each cell line and this study was undertaken as a pilot to test significance in change of response after subsequent irradiations and to inform future work.

## Materials and Methods

**Figure 1:**
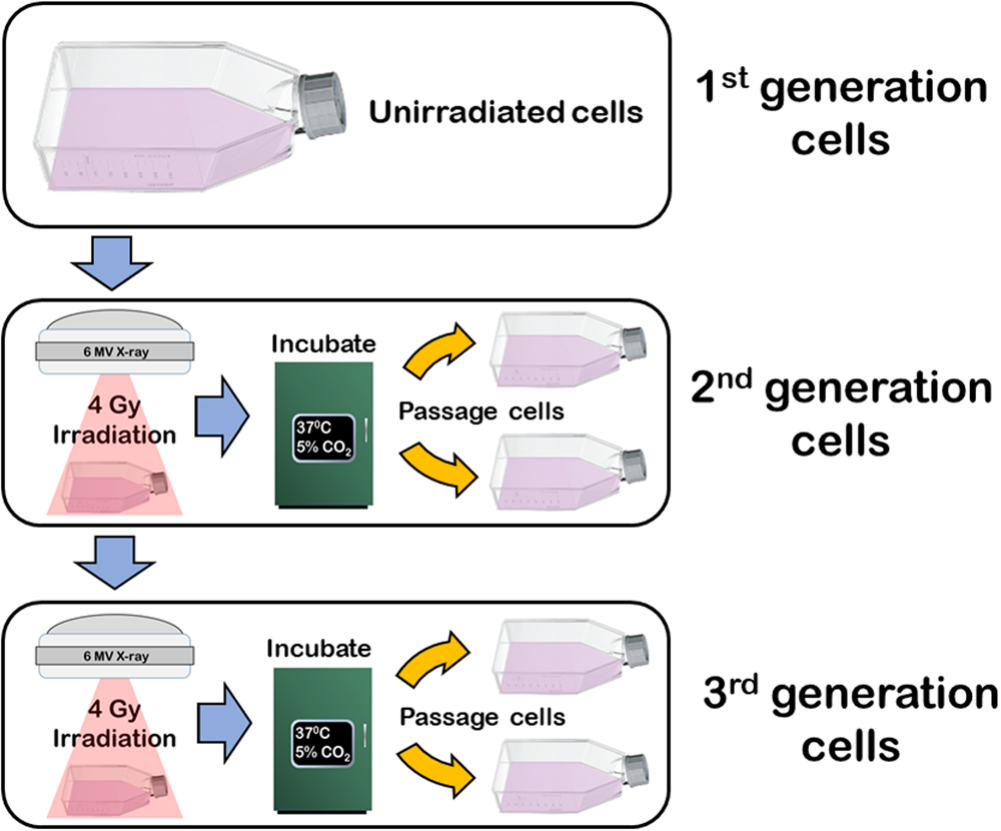
Generational development. Process to establish subsequent generations of each cell line by re-irradiation and culturing.

Changes in radiobiological responses in cell lines re-cultured after repeated exposures were termed ‘generational’ changes for the purposes of this study. Three discrete generations of the cell lines UM-SCC-1 (HPV negative) and UM-SCC-47 (HPV positive) were compared. Radiation dose to cell lines was measured as absorbed dose in gray (Gy), defined as the energy in joules deposited in mass by kilograms^19^. Original non-irradiated cultures were considered the 1^st^ generation of each cell line. Cell cultures irradiated once with 4 Gy X-ray irradiation, then re-cultured and passaged, became the 2^nd^ generation. Where 2^nd^ generation cultures were re-irradiated with 4 Gy, the re-cultured cells became the 3^rd^ generation (Fig. 1).

MTT assays were used in this study to assess resulting cell viabilities by metabolic activity in surviving populations after irradiation. MTT assays were performed as both cell lines proved to have low viability when seeded at low density for clonogenic assays and MTT assays facilitate quicker and higher throughput of sampling. Determinations of cell metabolic activity, as a function of dose, were compared between generations within cell lines and between cell lines by generation.

### Cell culture

Two head and neck squamous cell carcinoma cell lines were purchased from Merck Millipore (Darmstadt Germany) for this study. 1) UM-SCC-1 is a HPV negative cell line isolated from a recurrent tumour in the floor of mouth of a 73-year-old male. 2) UM-SCC-47 is a HPV positive cell line derived from a primary squamous cell carcinoma of the lateral tongue. Both cell lines originated from the laboratory of Dr. Thomas Carey, University of Michigan^20^. Cell cultures were grown in T75 flasks using RPMI 1640 cell growth medium (Sigma-Aldrich^®^ Darmstadt DE) supplemented with 10% foetal calf serum (FCS), 10 mM HEPES, 12.5 μg/ml penicillin and 16 μg/ml gentamycin. Cultures were grown in monolayer while incubated in humidified atmosphere at 37 °C with 5% CO_2_ and passaged after reaching exponential growth prior to confluency. Both cell lines were tested for the presence of mycoplasma (biotool.com B3903, Madrid ES) and found to be negative.

### Cell culture X-ray irradiation set up

**Figure 2:**
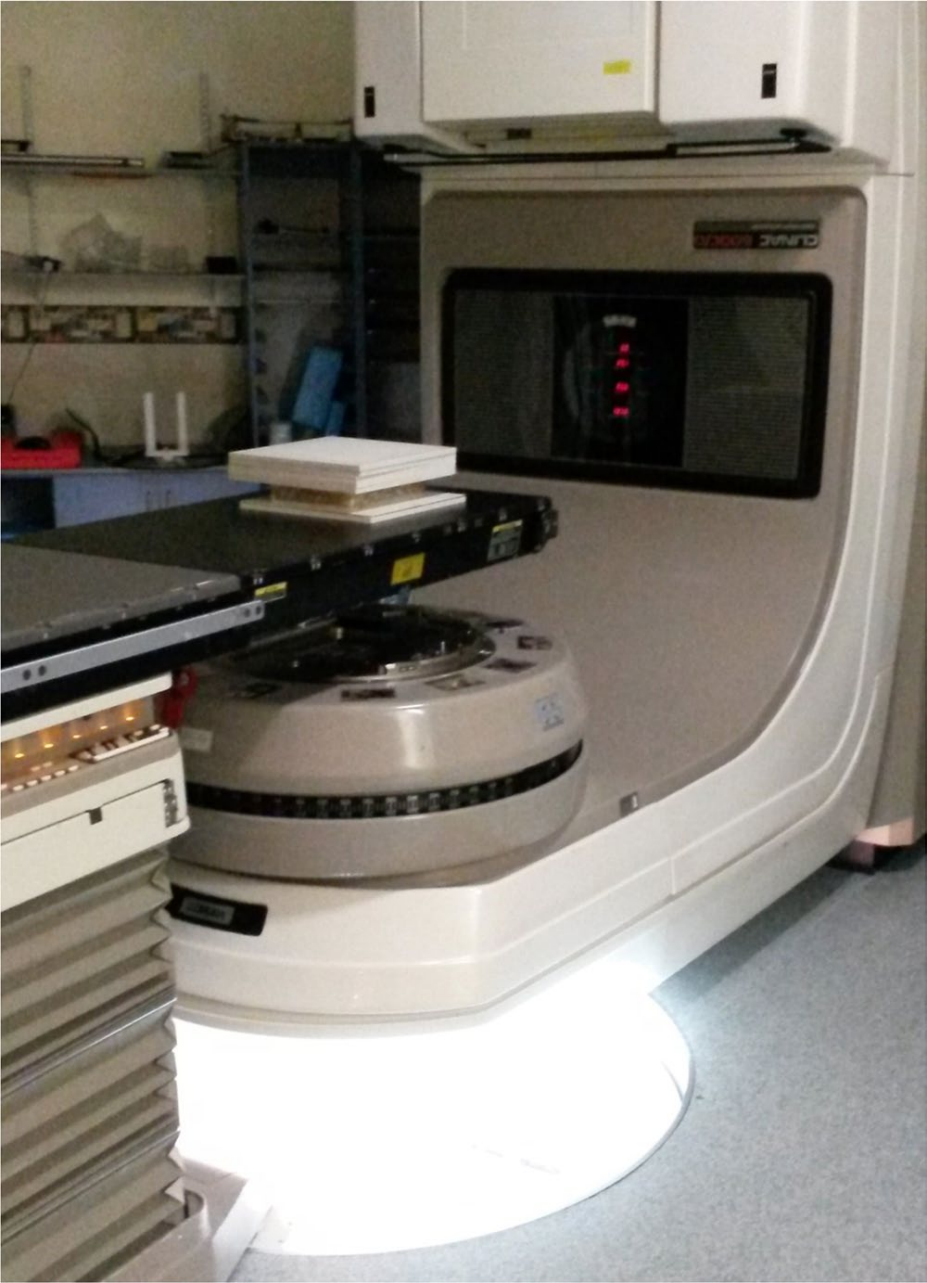
Irradiation setup for MTT experiments using a 6 MV beam from a Varian 600CD linear accelerator.

Cell cultures in T75 flasks were irradiated at the Radiation Oncology Department of the Royal Adelaide Hospital using a 6 megavolt (MV) X-ray beam from a Varian 600 C/D linear accelerator (Varian® Medical System, Palo Alto, CA). The linear accelerator was calibrated using IAEA TRS398 protocol^21^ and the radiation dose output checked on the day of irradiation with Daily QA 3™ device (Sun Nuclear, USA) before each radiation treatment. To achieve an electronic equilibrium at the cell monolayer, flasks were placed on top of a 13 mm slab of solid water (RW3; ρ = 1.0459 g/cm^3^ PTW, Freiburg Germany) with the table top at the level of the isocentre (Fig. 2) and irradiated from below with the gantry at 180°. A 20 × 20 cm radiation field size was used and flasks were encased in a wax block with a further 50 mm of solid water placed on top to provide full scatter conditions.

To investigate generational differences in metabolic activity as a function of dose, 3 generations of UM-SCC-47 were exposed to 1.0, 2.0, 4.0 and 6.0 Gy. As the UM-SCC-1 cells were more radioresistant, 3 generations of this cell line were exposed to 1.0, 2.0, 4.0, 6.0 and 8.0 Gy^15,22^. Sham irradiations of T75 flask cultures were performed as a control.

### MTT assay

MTT is a colourimetric non-clonogenic assay that measures cell viability in a culture by its metabolic activity. Briefly, cell cultures are stained with a yellow tetrazolium substrate (3-(4,5-dimethylthiazol-2-yl)-2,5-diphenyltetrazolium bromide) (MTT). Viable cells metabolise the substrate by reduction with NADH (Nicotinamide adenine dinucleotide) producing purple, water insoluble, formazan crystals. Dead cells do not reduce tetrazolium and thus the accumulation of purple colouration is proportional to metabolic activity and surviving cell viability^23^. Crystal solvents such as isopropanol or DMSO (Dimethyl sulfoxide) are used to homogenise the purple colouration. Spectrometry reading is automated by the use of plate readers which measure resulting optical density from formazan development by the absorbance of monochromatic light, typically around 570 nm. Absorbance values for each plate are indicative of the surviving metabolic activity for that culture. MTT assays are commonly used to measure cell viability post treatment (e.g. post irradiation) as they are comparatively quick and allow for high throughput analysis^23^.

To assay metabolic activity post irradiation, cells were trypsinised and centrifuged at 350 g for 5 minutes (Eppendorf 5810; Thermo Fisher Scientific, Waltham Mass.) then resuspended and counted by haemocytometer before replating in 96 well plates (Sigma-Aldrich^®^ Darmstadt DE). As MTT assays show a non-linear relationship between cell numbers and absorbance at high plating numbers, populations of 1.5 × 10^4^ cells per well (n = 10) were plated to be within linear function^24^. Staining and analysis was performed at 3 days post irradiation to allow sufficient time for apoptosis following irradiation. For each generation of both cell lines, 2 × 96 well plates were used. Five wells were seeded for each radiation dose in each plate, giving 10 wells of cells per radiation dose, plus control. Wells surrounding those seeded for culture were dosed with 150 ml PBS for humidification. The 96 well plates were incubated for 72 hours prior to staining with MTT, tetrazolium salts (3-[4,5-dimethylthiazol-2-yl]-2,5- diphenyltetrazolium bromide; thiazolyl blue) (Sigma-Aldrich^®^) and before cell populations reached confluency. At 72 hours, the cell medium was removed from the wells of adherent cells. 100 µL of 0.2 mg/ml tetrazolium salts was added to each well and incubated for 2 hours at 37 °C in 5% CO_2_ before checking formazan crystal formation by microscopy. The supernatant was removed from the wells and formazan crystals were dissolved by the addition of 200 µL isopropanol to each well to achieve homogenous optical density after 30 minutes. An additional column of empty wells received 200 µL of isopropanol to be read as blanks by microplate reader.

### Spectrophotometry

Optical density resulting from formazan development is proportional to surviving metabolic activity in each well. This was quantified by absorbency using FLUOstar Omega microplate reader (BMG LABTECH, Ortenberg, Germany). The optical density from blue-purple colouration in each well was measured as absorbance by the attenuation of 570 nm light. Absorbance was read in 10 wells for each dose (2 × 96 well plates) and for blanks where wells contained isopropanol only. Mean blank values for each plate were subtracted from absorbance values for each well.

### Statistical analysis

Absorbency data were analysed using Prism 7.01 (GraphPad Software, Inc. La Jolla, CA). Resulting absorbance values from 10-fold analysis of each dose were plotted as the ratio of mean absorbance to mean of control with standard error of the mean. Significance of results for intergenerational difference within cell lines were calculated by two-way ANOVA using Prism 7.01 and multiple comparisons within groups was performed using Tukey’s test. Significance of results between cell lines was calculated by multiple t test. Results of p < 0.05 were considered significant (*p < 0.05, **p < 0.01, ***p < 0.001).

### Data availability

Supporting data is available from the corresponding author upon reasonable request.

## Results

### Intergenerational results for cell lines

**Figure 3:**
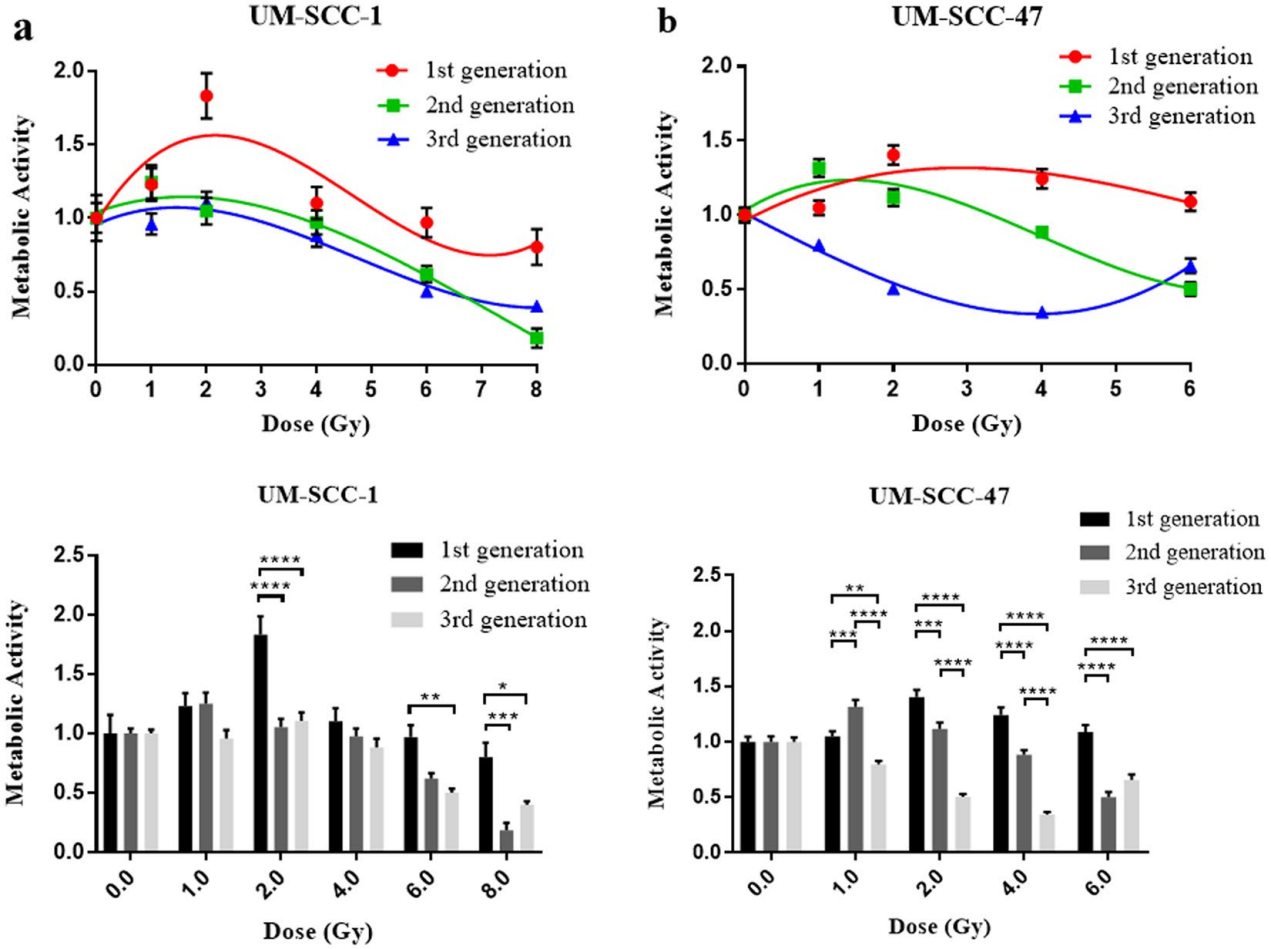
Results for surviving metabolic activity as a function of dose compared between 3 generations within cell lines. (**a**) UM-SCC-1. (**b**) UM-SCC-47, error bars show SEM.

MTT assay results for UM-SCC-1 showed greater metabolic activity in the 1^st^ generation than the control by way of radiation induced MTT effect, up to 4 Gy, which is explained further in the discussion. At higher doses, the 1^st^ generation continued to show greater metabolic activity than the latter two generations. The 2^nd^ and 3^rd^ generations show decreasing metabolic activity at >2 Gy, as shown in Fig. 3a. Little difference in surviving metabolic activity was seen between the 2^nd^ and 3^rd^ generations across the full range of radiation doses.

An even greater level of MTT effect was observed in UM-SCC-47 1^st^ generation. Metabolic activity higher than the control was seen across the dose range up to 6 Gy in the 1^st^ generation and up to 2 Gy in the 2^nd^ generation. An increasingly significant reduction in metabolic activity was observed however, in the 2^nd^ generation, against the 1^st^, at 4 and 6 Gy. The 3^rd^ generation of UM-SCC-47 showed even further reduction in metabolic activity by dose against the first 2 generations up to 4 Gy (Fig. 3b). At 6 Gy, metabolic activity returned to the level seen in the 2nd generation.

### Comparisons between cell lines by generation

**Figure 4:**
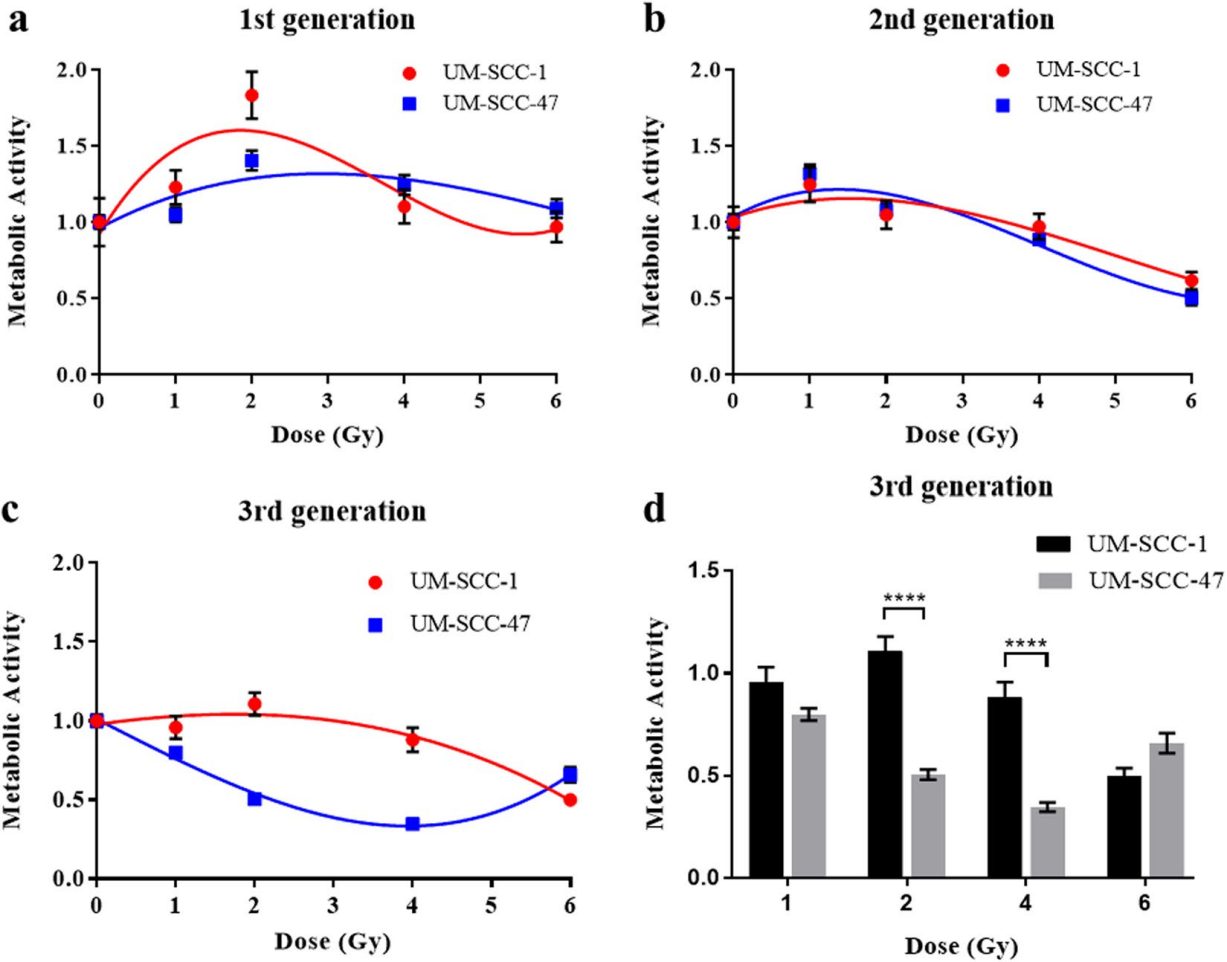
Changes in surviving metabolic activity compared between cell lines by generation. (**a**–**c**) Surviving metabolic activity in the 1^st^, 2^nd^ and 3^rd^ generations respectively, for both cell lines as a function of dose. (**d**) Significance of difference between cell lines at 2 and 4 Gy, error bars show SEM.

The 1^st^ generations of both UM-SCC-1 and UM-SCC-47 showed radiation induced MTT effect as mentioned above. Enhanced levels of formazan development, above that of the untreated cells, was observed across the dose range to 6 Gy. UM-SCC-1 showed the most pronounced effect at 2 Gy. The 2^nd^ generation of each cell line displayed very little difference in MTT responses across all X-ray doses and resulting trend lines as a function of dose were very similar (Fig. 4b). The only significant differences in MTT response between cell lines by generation were seen in the 3^rd^ (Fig. 4c) where UM-SCC-47 showed a continuing decline in surviving metabolic activity after subsequent irradiations in the 2–4 Gy dose range. Here, the surviving metabolic activity of the 3^rd^ generation in UM-SCC-47 was only 46% and 39% of the level in UM-SCC-1 at 2 and 4 Gy respectively.

## Discussion

In this study, radiation induced changes in the radiobiological response of the two HNSCC cell lines were investigated by surviving metabolic activity. Progressive change in radiosensitivity was examined by intergenerational comparisons within cell lines as well comparisons between cell lines by generational response. Measures of resulting cell viability, by surviving metabolic activity as a function of X-ray dose, show changing radiosensitivity between generations and between cell lines. The extent of metabolic activity in each cell culture, 3 days after irradiation, was dependent on its ability to evade the lethal effects of radiation, potentially repair damaged DNA, and express continuing metabolic activity. Metabolic activity, post irradiation was measured by MTT assay. This process quantifies the surviving metabolic activity of cells by mitochondrial reduction of a yellow tetrazolium salt solution to insoluble formazan crystals by the enzyme succinate dehydrogenase. Formazan development does not occur in dead cells and, 3 days post irradiation, the optical density arising from the blue-purple pigmentation of formazan, provides a quantification of surviving metabolic activity^25^.

Changes in generational responses in the HPV negative UM-SCC-1 are only evident between the 1^st^ and 2^nd^ generations. The subsequent re-irradiation, following re-culture of the 2^nd^ generation to develop the 3^rd^, was observed to have little effect on radiosensitivities when comparing between these generations. HPV positive UM-SCC-47 responded differently to subsequent exposures where each generation showed increasing radiosensitivity with re-irradiation. Surviving metabolic activity was generally lower as a function of dose for both cell lines with the exception of the 1^st^ generation where radiation induced MTT effect resulted in an elevation of metabolic response greater than the untreated cells. The first 2 generations of both cell lines show elevated metabolic activity over that of the control up to 2 Gy. This enhanced reduction of tetrazolium salts by irradiated cells has been reported in literature as radiation induced MTT effect by Blockhuys *et al*.^26^ finding elevated formazan concentration in cells at doses between 1 and 6 Gy. Their investigation of this effect found it was not due to elevated cell numbers but rather increased metabolic reduction of tetrazolium facilitated by enhanced plasma membrane permeability. Plasma membranes exposed to X-rays are affected by reactive oxygen species (ROS) causing membrane disruption resulting from lipid peroxidation^3^. It was noted in this study that this effect was most pronounced in the 1^st^ generations of both UM-SCC-1 and UM-SCC-47 and was seen only at lower doses in the 2^nd^ generations. Optical densities from MTT assay, greater than the controls, may possibly demonstrate greater radiobiological effect on cell membrane in the previously unirradiated cells, at this dose range, than cellular lethality and loss of metabolic function^26^.

Although clonogenic assays remain the gold standard for determining radiosensitivity by surviving fractions from irradiated cells, it has the drawbacks of lengthy incubation times and difficulties arise with non-colony forming cell lines^24^. In this investigation MTT assays were used as both UM-SCC-1 and UM-SCC-47 showed poor plating efficiencies and proved unsuitable for clonogenic assay. It is important to note that the MTT assay does not specifically measure clonogen survival but rather the viability of all cells in a culture irrespective of differentiation status. MTT assays do however have the advantage of not requiring colony formation at low plating densities and, being a semi-automated process, enable high volume throughput and easier repeat experimentation.

Metabolic activity results showed significant alterations in surviving function, for dose, between generations within each cell line. The 3^rd^ generation of UM-SCC-47, demonstrated depressed metabolic activity post irradiation, after passage and re-culture following 2 previous exposures of 4 Gy, compared to 1^st^ and 2^nd^ generations. The observed changes in cellular responsiveness to dose indicate increasing radiosensitivity with subsequent irradiation at 4 Gy. Curiously, the 3rd generation of UM-SCC-47 also showed and elevated response at 6 Gy which matched that of the 2nd generation.

Significant change was only observed in UM-SCC-1 between the 1^st^ and 2^nd^ generations, possibly due to the radiation induced MTT effect in the 1^st^ generation. Unlike UM-SCC-47, there was no further increase in radiosensitivity with the 3^rd^ generation of UM-SCC-1. This resulted in significant differences observed in radiosensitivity between the 2 cell lines in the 3^rd^ generation. Here the HPV positive cell line showed a continuing increase in radiation sensitivity in contrast to the HPV negative cell line. This is of interest given better clinical outcomes observed for HPV positive HNSCC and illustrates a distinction between the cell lines that may be related to their HPV status. An investigation by Kimple *et al*.^27^ of 4 HPV positive and 4 HPV negative cell lines (including UM-SCC-1 and 47) found all HPV positive cell lines more radiosensitive than the HPV negative lines. Radiosensitivity was assessed by the surviving fraction of cells after a 2 Gy exposure (SF_2_). The mean SF_2_ for the HPV positive status cell lines was 22% compared to the negative status SF_2_ of 59%. On a molecular basis, studies examining differences in radiosensitivity between HPV positive and negative HNSCC, investigated the development and decay of the γH2AX molecule where γH2AX form detectable foci at DNA double strand breaks (DSB) resulting from ionising radiation. The extent to which these foci are resolved is indicative of cellular DNA repair^28^. HPV positive cell lines have demonstrated significantly greater levels of γH2AX foci, persisting after 24 hours, than HPV negative cell lines, suggestive of poorer DNA repair and consequently greater radiosensitivity. Elevated γH2AX foci were also found to correlate with G2 arrest and share an inverse relationship with cell survival^15,29^. While differential mechanisms of radiosensitivity between HPV positive and negative aetiologies remain largely unclear, such investigations by cell line, however small, can direct future work.

The radiobiological differences in HNSCC cell lines, according to HPV status, demonstrated in our study and others, are consistent with the better clinical outcomes observed for HPV positive HNSCC. These results should encourage further work to identify the mechanisms underlying the observed differences in radiobiological responsiveness between HPV positive and negative HNSCC cell lines. This information will be important because clinical trials are currently underway to investigate reductions in the intensity of therapeutic regimens employing radiotherapy in HPV positive HNSCC^15^.

## Conclusions and Future Work

In this study, we have used a novel generational approach to demonstrate altered cellular responsiveness to irradiation in the two HNSCC cell lines investigated, after repeated X-ray exposures. Our findings of altered to radio-responsiveness by subsequent exposures and re-culturing of cell lines show generational increases in radiosensitivity. Although increasing radiosensitivity in UM-SCC-1 cells was not apparent after the 2nd generation, it was observed to continue for the HPV positive UM-SCC-47 cells. Unlike dose fractionation schedules applied clinically, our data do not indicate that repeated irradiation of HNSCC cell lines resulted in increased radioresistance. These results however must be viewed in light of their limitations where the surviving cultures, examined after 3 days, are irrespective of the clonogenic population which has greater bearing on post-radiotherapy tumour re-growth. To this end, clonogenic assays are required in future work to examine the generational behaviour of clonogens in response to subsequent irradiations. The conclusions of this study, in respect of differences between HPV positive and HPV negative cell lines, are limited by the small representation of cell lines. Future work requires extended representation by greater number of cell lines of each HPV status to determine radiobiological behaviour common to each status. Additionally, the development of further generations of each cell line needs to be undertaken to show more extensively radiobiological change in cell lines following fractionated exposures. Ultimately, the generational technique developed and observations of significant differences in radiobiological responses in this work need to be investigated and validated using tumour biopsies taken from tumours of different origins and stages.
